# Endophytic ability of the insecticidal bacterium *Brevibacillus laterosporus* in Brassica

**DOI:** 10.1371/journal.pone.0216341

**Published:** 2019-05-22

**Authors:** M. Marsha Ormskirk, Josefina Narciso, John G. Hampton, Travis R. Glare

**Affiliations:** Bio-Protection Research Centre, Lincoln University, Lincoln, New Zealand; Arizona State University, UNITED STATES

## Abstract

*Brevibacillus laterosporus* (*Bl*), is an insecticidal bacterium recorded as toxic to a range of invertebrates after ingestion. Isolates of *Bl*, which were initially recovered from surface-sterilised cabbage (*Brassica oleracea* var. *capitata*) seeds, were able to colonise brassica plants in the laboratory and field. The bacterium was recovered from surface-sterilised leaf, stem and root sections of seedlings after inoculation with *Bl* vegetative cells under laboratory conditions, and from mature cabbage plants sprayed with *Bl* in a field trial. The identity of the recovered bacterial isolates was confirmed by PCR through amplification of 16S rDNA and two strain-specific regions. The effect on diamondback moth (DBM) insect herbivory was tested with cabbage seedlings treated with one isolate (*Bl* 1951) as the strains are toxic to DBM after direct ingestion. While no effect on DBM larval herbivory was observed, there was a significant reduction of DBM pupation on the *Bl* 1951 colonised plants. The presence of *Bl* 1951 wild type cells within cabbage root tissue was confirmed by confocal microscopy, establishing the endophytic nature of the bacterium. The bacterium was also endophytic in three other brassica species tested, Chinese kale (*Brassica oleracea* var. *alboglabra*), oilseed rape (*Brassica napus* var. *oleifera*) and radish (*Raphanus sativus*).

## 1. Introduction

*Bl* is an aerobic, spore-forming bacterium. Some strains of *Bl* have been identified as potential bio-control agents of a range of invertebrate pests, with isolates reported to have toxicity against insects belonging to the orders of Lepidoptera, Coleoptera and Diptera, against the mollusk *Biomphalaria glabrata* and nematodes [1, 2]. In New Zealand, isolates of *Bl* have been discovered with toxicity to larvae of the diamondback moth (DBM) (*Plutella xylostella*) [3]. This insect species is considered to be one of the most damaging insect pests of cabbages and other brassicas [4].

*Bl* strains have also shown activity against plant pathogens and other microbes. Chen et al. [5] used a strain which was able to colonise the plant rhizosphere to reduce the impacts of potato common scab (PCS), caused by *Streptomyces* spp. Another rhizosphere colonising strain was active against brown stripe of rice caused by *Acidovorex avenae* subsp. *avenae* [6]. Antifungal effects have also been seen with other strains of *Bl* [7, 8]. Endophytic bacteria belonging to the genus *Brevibacillus* have been isolated and identified in crops such as *Sphaerophysa salsula*, a wild legume in China [9], in tomato [10], alfalfa [11], common bean [12], rice [13], coffee [14], banana [15], and in lichens [16]. However, there are no known reports of *Bl* as an endophyte. The mode of action of the New Zealand *Bl* isolates toward the DBM is still being investigated but appears to be toxic rather than infective. Given the original recovery of the isolates from surface-sterilised cabbage seeds, it was hypothesised the bacterium may be capable of endophytic growth. There are several advantages if the bacteria are capable of living endophytically in economically important brassica crops subject to DBM attack. For example, if the strains are capable of conferring protection against DBM larval herbivory when occurring as endophytes in brassica crops, it may substantially reduce the need for foliar applications of the spores, potentially reducing control costs.

The main question addressed in this study was whether the New Zealand *Bl* isolates 1821 and 1951 were capable of living endophytically within cabbage plants. This hypothesis was tested in the laboratory and through a field trial. The *Bl* isolates were evaluated for their ability to live endophytically in seedlings of four *Brassica* species, cabbage, Chinese kale, oilseed rape and radish. Additionally, the effect of cabbage seedlings treated with *Bl* 1951 as a potential endophyte on DBM larval herbivory was tested in three separate bioassays. Lastly, the presence of *Bl* 1951 in cabbage root tissue was examined by confocal microscopy.

## 2. Materials and methods

### 2.1 Strains and culture conditions

*Bl* 1821 and 1951 were isolated from New Zealand grown brassicas in New Zealand in 2008 [3]. Strain 1821 is deposited as V12/0019 and isolate 1951 deposited as V12/001945 in the National Measurement Centre, Melbourne, Australia. Cultures were routinely grown in sterile Luria-Bertani (LB, Difco) agar (LBA) at 30°C. Additionally, cultures were grown in sterile Luria-Bertani Miller broth (LB, Difco) (LBB) at 250 rpm and 30°C overnight (O/N) for ~16–18 hours. These cultures were designated as O/N cultures.

To grow spores of *Bl* 1821 and 1951, 100 μl of O/N culture was inoculated in 25 ml modified LBB, modified from Zeigler [17], and designated mLB^+^ (7.7 mM K_2_HPO_4_, 42 mM KH_2_PO_4_, 2.5% w/v LB, 0.0125% w/v NaOH, 5.25 mM NTA, 0.59 mM MgSO_4_, 0.91 mM CaCl_2_, 0.04 mM FeSO_4_, 2.5 mM MnCl_2_ and 1% w/v glucose; pH 7.6), in a 250 ml flask, or 100 ml mLB^+^ in a 1 L flask to allow for aeration at 250 rpm and 30°C for six to seven days.

### 2.2 Cabbage seed surface sterilisation and seed germination in Murashige and Skoog agar medium

Initial experiments were conducted using cabbage (*Brassica oleracea* var. *capitata*). Seeds were washed by soaking with slight shaking in 0.01% Tween-80 for one minute then rinsing in sterile water for one minute. Subsequently, the seeds were surface-sterilised in 2% (v/v) of 53 g/L sodium hypochlorite (MaxKleen, Pure Hospital Grade Sanitizing Bleach) for three minutes. Finally, the seeds were sterilised for one minute in 70% ethanol (EtOH) and subsequently washed three times with sterile Milli Q water (MQW). The surface-sterilised seeds were sown on ½ Murashige and Skoog (MS) media (Sigma, Life Sciences), containing 0.8% agar; pH 7. The seeds were incubated for seven days at room temperature with a 12:12 hour light-dark cycle prior to seedling inoculation.

### 2.3 Recovery of *Brevibacillus laterosporus* 1821 and 1951 from cabbage seedlings over time

#### Inoculation of cabbage seedlings with *Brevibacillus laterosporus* 1821 and 1951

Seven-day-old cabbage seedlings, grown from surface-sterilised seeds as described above, were inoculated with *Bl* 1821 and 1951 cells by dipping the roots in 10 mL of an O/N culture. The cell concentration of the *Bl* 1821 and *Bl* 1951 O/N cultures was 1.3 x 10^9^ cells/mL and 2.2 x 10^9^ cells/mL, respectively. Subsequently, the inoculated seedlings were transplanted to a 50 mL Falcon tube containing 40 mL equivalent of gamma-irradiated soil and 15 mL of sterile MQW. An additional 500 μl of O/N culture was added to the soil of the transplanted seedlings. Sterile LBB was used as a negative control. The plants were placed in a sterile plastic cereal container (Sistema Klip It Blue Cereal Storer, 4.2 L). The experiments contained three treatments: 1) Negative control, LB; 2) *Bl* 1821; and 3) *Bl* 1951. Each treatment included four plants per replicate with three replicates (12 plants per treatment; S1A Fig). Inoculated cabbage plants were incubated at a constant temperature of 22°C, a 12:12 hour light-dark cycle and 65% humidity in a Panasonic Versatile Environmental Test Chamber MLR 352H.

#### Sampling and surface sterilisation of cabbage plants inoculated with *Brevibacillus laterosporus* 1821 and 1951

One randomly selected plant per replicate of each treatment was harvested each week for a total of three samples per treatment per week (S1A Fig). The soil was washed off the roots with MQW and 70% EtOH before surface sterilisation. Surface sterilisation started with a one-minute wash in 0.05% Tween-80, followed by a brief rinse in sterile MQW. Next, the samples were washed for three to five-minutes in 2% (v/v) of 53 g/L sodium hypochlorite (MaxKleen, Pure Hospital Grade Sanitizing Bleach) containing 0.05% Tween-80. Finally, the samples were washed briefly in sterile MQW, followed by a one-minute wash in 70% EtOH and then washed twice for one minute in sterile MQW. Samples were cut into pieces and placed onto semi-selective LBA containing 0.02 mg/mL streptomycin (LBA+strept). Each agar plate was divided into three parts to accommodate all surface-sterilised parts of one plant: the roots, stems and leaves. The plates were incubated at 28°C for two to three days until bacterial colonies emerged from the plant tissue.

#### Identification of *Brevibacillus laterosporus* 1821 and 1951 from bacterial colonies recovered from surface-sterilised cabbage seedlings

Bacterial colonies from the semi-selective agar plates were grown O/N as described previously. DNA was extracted from 1 mL of O/N culture using the Blood N’ Tissue DNA extraction kit (Qiagen). The bacterial species were identified by the amplification of the 16S rDNA region using universal primers: F8 forward primer, AGTTTGATCCTGGCTCAG, and the r1510 reverse primer, GGTTACCTTGTTACGACTT [18]. The PCR cycle was four minutes at 95°C for one cycle followed by 15 seconds at 94°C, 20 seconds at 55°C and three minutes at 72°C for five cycles. Subsequently, the samples were run for 15 seconds at 94°C, 20 seconds at 50°C and three minutes at 72°C for 35 cycles. Finally, the DNA was heated for four minutes at 72°C.

The PCR products were sequenced (Sanger sequencing, Lincoln University Sequencing Unit) and forward and reverse sequences aligned using pairwise Multiple Sequence Comparison by Log-Expectation (MUSCLE) in Geneious version 8.1.7. The *Bl* 1821 and 1951 consensus sequences were aligned with 16S rDNA sequences from other *Bl* strains, other *Brevibacillus* and *Bacillus* species from the NCBI nucleotide database (NCBI, 2017). Phylogenetic trees were built using the Tamura-Nei genetic distance model and the Neighbour-Joining tree building method. Rooted trees were constructed by using *Bacillus thuringiensis* subsp. *kurstaki* as an outgroup. Bootstrapping was used as the resampling method. A consensus tree was created from 1000 replicates, and the support threshold was set at 50%.

### 2.4 The quantification of *Brevibacillus laterosporus* 1951 from inoculated cabbage plants over time

Seven-day-old cabbage seedlings (cv. Axiros NS), grown from surface sterilised seeds as described in Section 2.2, were inoculated by root dipping with O/N culture of 1951 and sterile LBB as the control following the steps described in Section 2.3. The number of plants was the same as in the previous experiments. Using the semi-selective LBA+stept+cyclo medium (LBA with 0.02 mg/mL streptomycin and 0.125 mg/mL cycloheximide), plants were sampled to determine the quantity of *Bl* 1951 in CFU/mL on a weekly basis starting at seven days after inoculation (DAI) until 28 DAI. The length and diameter of the leaves and stems and the length of the roots were recorded before surface sterilisation using a ruler and a caliper. Cabbage plants were surface sterilised as described above. The leaf, stem and roots of the surface-sterilised plants were separated and cut into smaller pieces using a sterile scalpel. The cut pieces of each plant part were placed in a 2 mL tube with a sterile metal bead to which 1 mL of sterile 0.9% NaCl solution was added. Homogenisation of each plant part was done for 30 seconds at 1500 rpm using a bead beater machine (1600 MiniG, SPEX Sample Prep). The tubes were inverted to mix the solution, and the homogenisation was repeated for another 30 seconds. The debris from the homogenised solution was filtered by pipetting the solution into a 1.7 mL tube, which was lined with sterile cotton wool, and placed on top of a 2 mL tube.

Serial dilutions of 1:10, 1:100 and 1:1000 of the full strength homogenised solutions were prepared during the first sampling at 7 DAI. Serial dilutions of 1:10 and 1:100 were also prepared at the other three sampling dates to allow better bacterial recovery. A volume of 100 μl of each dilution and the full strength (undiluted) was pipetted onto the surface of the semi-selective medium (LBA+strep+cyclo). Two plates were inoculated with full strength and diluted solutions per plant part per dilution and incubated at 25°C. Bacterial colonies growing in the medium were counted five to seven days after plating. The CFU/mL was computed as the number of colonies per plate/volume of solution inoculated per plate (0.1 mL).

### 2.5 Inoculation of other brassica species with *Brevibacillus laterosporus* 1951

The same procedures used in Section 2.2 with the cabbage seedlings were followed for seed sterilisation and inoculation of seedlings belonging to three different brassica species; Chinese kale (*Brassica oleracea* var. *alboglabra*, Musashino Seed Co.), oilseed rape (*Brassica napus* var. *oleifera* cv. Goliath) and radish (*Raphanus sativus* cv. Red Cherry). The experiments were conducted separately for each brassica species. Each experiment contained two treatments; plants inoculated with an O/N culture of *Bl* 1951, and plants inoculated with sterile LB broth as the negative control. The experimental design and growth conditions were as described in Section 2.3.

#### Detection of *Brevibacillus laterosporus* 1951 from surface-sterilised brassica seedlings

Brassica seedlings were surface sterilised and incubated as described in Section 2.3. As a control for successful surface sterilisation, both sides of surface-sterilised seedling samples were lightly pressed onto a LBA control plate before the samples were cut into smaller pieces. Surface-sterilised samples were discarded if any colonies grew from the corresponding control plates. Aside from the cabbage samples, the surface sterilised samples of the other brassica species were incubated on LBA+strept+cyclo instead of LBA+strept. The cabbage samples were incubated as described in Section 2.3, while the other species were incubated at 25°C for five to seven days. The difference in temperature was due to incubator availability. Only one with a set temperature of 25°C was available for the later experiments. The increased incubation time was to compensate for the lower temperature.

Bacterial colonies from the semi-selective agar plates were grown O/N in LBB as described previously. The next day the O/N cultures were used for DNA extraction using a 5% Chelex 100 resin (Bio-Rad). DNA extraction was conducted according to the manufacturer’s guidelines. To ensure the identity of the strain, specific primers were used for *Bl* 1951 that targeted a *cry*35-like gene using the forward primer (ATGTCCATAAATATAGATCCTTCA) and the reverse primer (ACACACTTTCGAAATATGAGG). The expected size of the gene product was 1107 bp. The PCR conditions were: 95°C for 5 min followed by 40 cycles of denaturation at 95°C for 45 seconds, annealing at 60°C for 45 seconds, primer extension at 72°C for two minutes and a final extension at 72°C for 7 minutes. PCR-products were visualised on a 1% agarose gel.

### 2.6 Bioassay of inoculated plants against diamondback moth larvae

Cabbage plants in each of the three repeat bioassays were grown and inoculated following the procedures as described in Sections 2.2 and 2.3, using cabbage plants inoculated with vegetative cells of the O/N cultures of *Bl* 1951 and sterile LBB as the negative control. Leaves were harvested from *Bl* 1951 inoculated and non-inoculated control plants at 11 DAI (day after inoculation), 16 DAI and 20 DAI, for the first, second and third bioassay, respectively. Leaves from inoculated and control plants were fed to the DBM larvae. All leaves except for the very small ones were cut off the plant using a sterile scalpel. Each leaf was then placed in a 35 mL plastic container (Huhtamaki) lined with 30 mm filter paper, which was moistened with 100 μl of sterile water. Three third instar larvae were placed on top of the leaf using a fine camel brush. The experiment was replicated three times with five leaves per replicate. The filter paper discs were replaced and a new leaf was fed to the larvae daily until seven days after initial feeding (DAF). The leaves were placed in an incubator at 25°C and 16:8 hour light/dark cycle. The number of live, dead and pupating larvae was recorded daily for seven days using a stereomicroscope to examine larvae. The number of larvae that pupated was recorded starting at four DAF until seven DAF. The percentage pupation was computed as: number of pupae/total number of larvae x 100. A mean comparison between the control and *Bl* 1951 was performed by an Analysis of Variance (ANOVA) using Genstat v.18 (VSNi), followed by a 5%-level one-sided least significance difference (LSD) test of the difference between the treatments. A one-sided test was justified by the fact that the bacterium performed better than the control in previous insect bioassays.

### 2.7 Field study permits for field trial

The field study was performed under the HSC100108 license, granted by the Environmental Protection Authority (EPA) of New Zealand, and conducted on the privately owned land of Lincoln University at the Field Service Centre. The field trial was set up as part of the Next Generation Bio-Pesticides programme (Ministry for Business, Innovation and Employment (MBIE), NZ- programme C10X1310.

#### Recovery of *Brevibacillus laterosporus* 1951 from field-treated plants

Plants were sampled from a field trial that was comparing three potential biological control agents of cabbage pests, including *Bl* 1951. A randomised block design was set up containing eight treatments and eight blocks. Each treatment plot contained four plants in total (S1 Fig). This field trial was used as an opportunity to sample treated plants to detect endophytic colonization by *Bl* 1951 in adult cabbage plants sprayed with *Bl* 1951 spores. Cabbage plants (cv. Derby Day) were sprayed fortnightly up to three times during approximately ten weeks, from seedling until full growth, with ~1500 L/Ha of 7 x 10^8^ to 2 x 10^9^ spores/mL of *Bl* 1951 containing 0.5% v/v Synoil adjuvant surfactant (Orion AgriScience). The negative control included plants that were sprayed with water containing 0.5% of Synoil adjuvant surfactant.

Twenty-four 10-week old plants were sampled for the presence of *Bl* 1951 as an endophyte. Sixteen plants were sampled from the *Bl* 1951 field trial plots and eight plants were sampled from the negative control plots (S1B Fig). From each plant, an inner leaf, outer leaf and root segment was sampled. The base of the midvein/midrib of each leaf was sampled using a 3 cm diameter core borer. All plant samples were washed with tap water and gently wiped with 70% EtOH prior to surface sterilisation. Surface sterilisation and incubation of the samples was as described in Sections 2.3 and 2.5 with three modifications: 1) Five to seven-minute incubation in 3% to 4.5% (v/v) of 53 g/L sodium hypochlorite (MaxKleen Pure Hospital Grade Sanitizing Bleach), containing 0.01% Tween-80; 2) One to three minute incubation in 70% EtOH; 3) Cut samples were placed onto LBA+stept+cyclo.

Specific identification of *Bl* 1951 was conducted as described in section 2.5. A *cry*27-like gene was targeted in addition to the *cry*35-like gene for a more sensitive detection of *Bl* 1951 as the strain contains both genes. The forward and reverse primer sequences for the *cry*27-like gene were ATGAATAAAAATGACAAAAATAAG, and GTACGACTCAACCGGAAT, respectively, with an expected gene product size of 1970 bp.

### 2.8 Microscopic observation of *Brevibacillus laterosporus* colonisation inside the cabbage tissues

Cabbage seedlings, grown from surface sterilised seeds as described in Section 2.2, were inoculated with O/N culture of *Bl* 1951 and sterile LB broth as the negative control following the steps described in Section 2.3. A seven-day-old *Bl* 1951 sporulating culture, grown in LB broth at 30°C and 250 rpm, was used as a positive control. Plants were harvested at 7, 14 and 21 DAI by carefully pulling out the plants from the tubes. The roots were cleaned of soil and other debris by washing in running tap water and placed in 50 mL tubes. The plants were then fixed and stained following the protocol by Ramonell et al. [19] with modifications. Congo Red at the final concentration of 10 μg/mL and Phloxine B at 100 μg/mL were used to stain the plant tissues and *Bl c*ells, respectively. Phloxine B is reported to be an effective differential bacterial stain for bright field, fluorescence and confocal microscopy observations [20]. The stained plant samples were viewed with 561 nm excitation wavelength for Congo Red and 488 nm for Phloxine B using a LSM 510 META (Zeiss) laser scanning microscope at 63,000 X magnification.

## 3. Results

### 3.1 Recovery of *Brevibacillus laterosporus* 1821 and 1951 from cabbage plants

**Fig 1:**
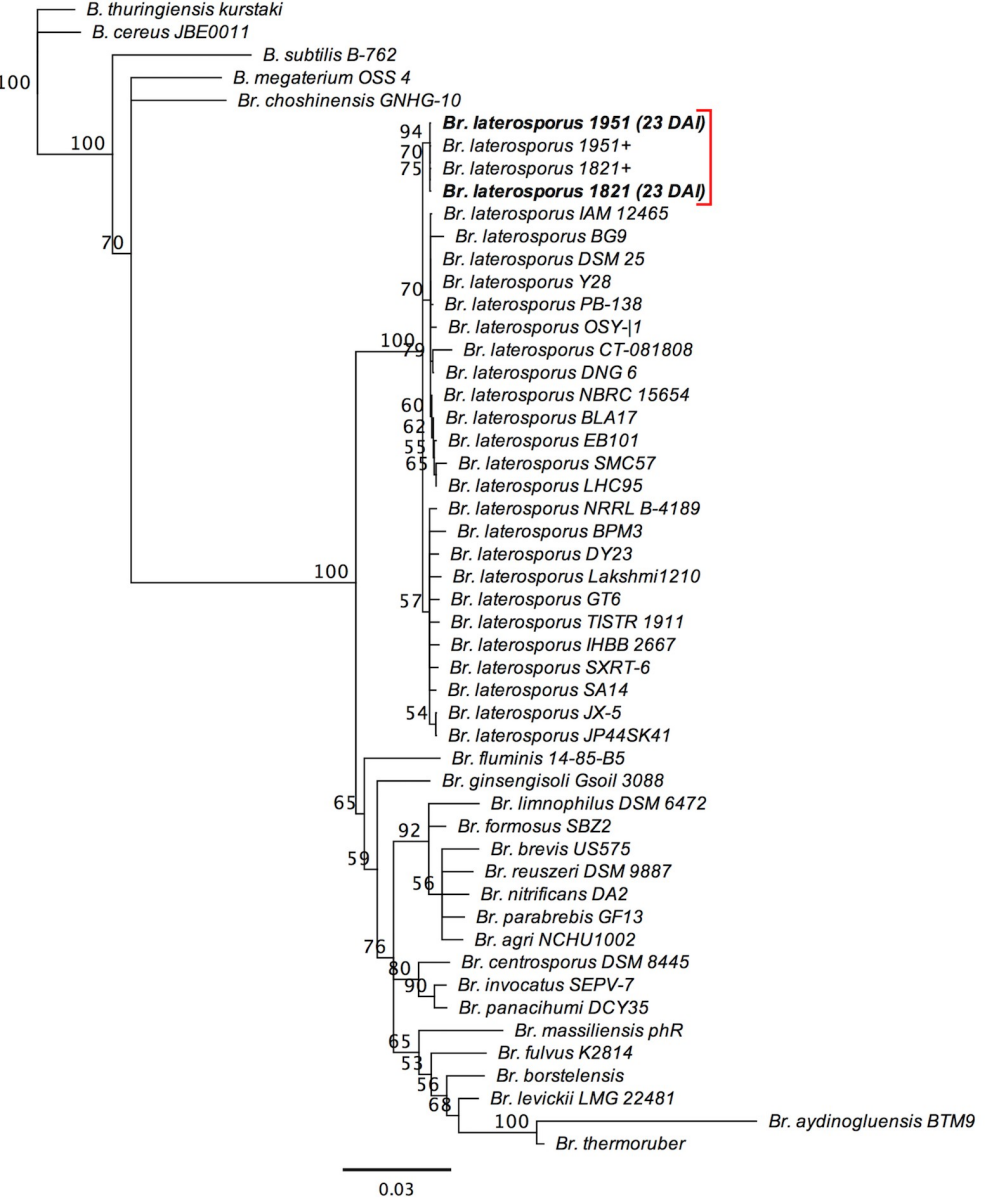
Phylogenetic tree of 16S rDNA sequences of the *Brevibacillus laterosporus* 1821 and 1951 samples recovered from cabbage seedlings 23 days after incubation. Formatted in Geneious [21]. The scale bar represents the nucleotide substitutions per site (the number of changes per 100 nucleotide sites). The red bracket indicates the position of the *Bl* 1821 and 1951 potential endophyte samples and the *Bl* 1821 and 1951 16S rDNA derived from the stock cultures as positive controls (+). Abbreviations: B. = *Bacillus*; Br. = *Brevibacillus*.

*Bl* was recovered from treated cabbage plants in laboratory experiments. Colonies grew from surface-sterilised samples from the roots, stems and leaves 28 days after inoculation (DAI). Bacterial colonies were also observed in samples of the negative control at seven DAI (S2 Fig), and identified as *Bl* by 16S rDNA sequencing (S3 Fig). This may have been caused by inadequate sterilisation of the scalpel that was used to cut the surface-sterilised plants into parts before inoculation on selective agar for the first sampling period. After modifying the technique, no colonies emerged from the negative control in the following weeks, 14 DAI, 23 DAI and 28 DAI (S4, S5 and S6 Figs). All endophytic DNA samples were identified as *Bl* by 16S rDNA sequencing (Fig 1). These results suggest that *Bl* 1821 and 1951 are capable of endophytic growth in cabbage seedlings up to 28 DAI.

### 3.2 Quantification of *Brevibacillus laterosporus* 1951 from inoculated cabbage plants

**Fig 2:**
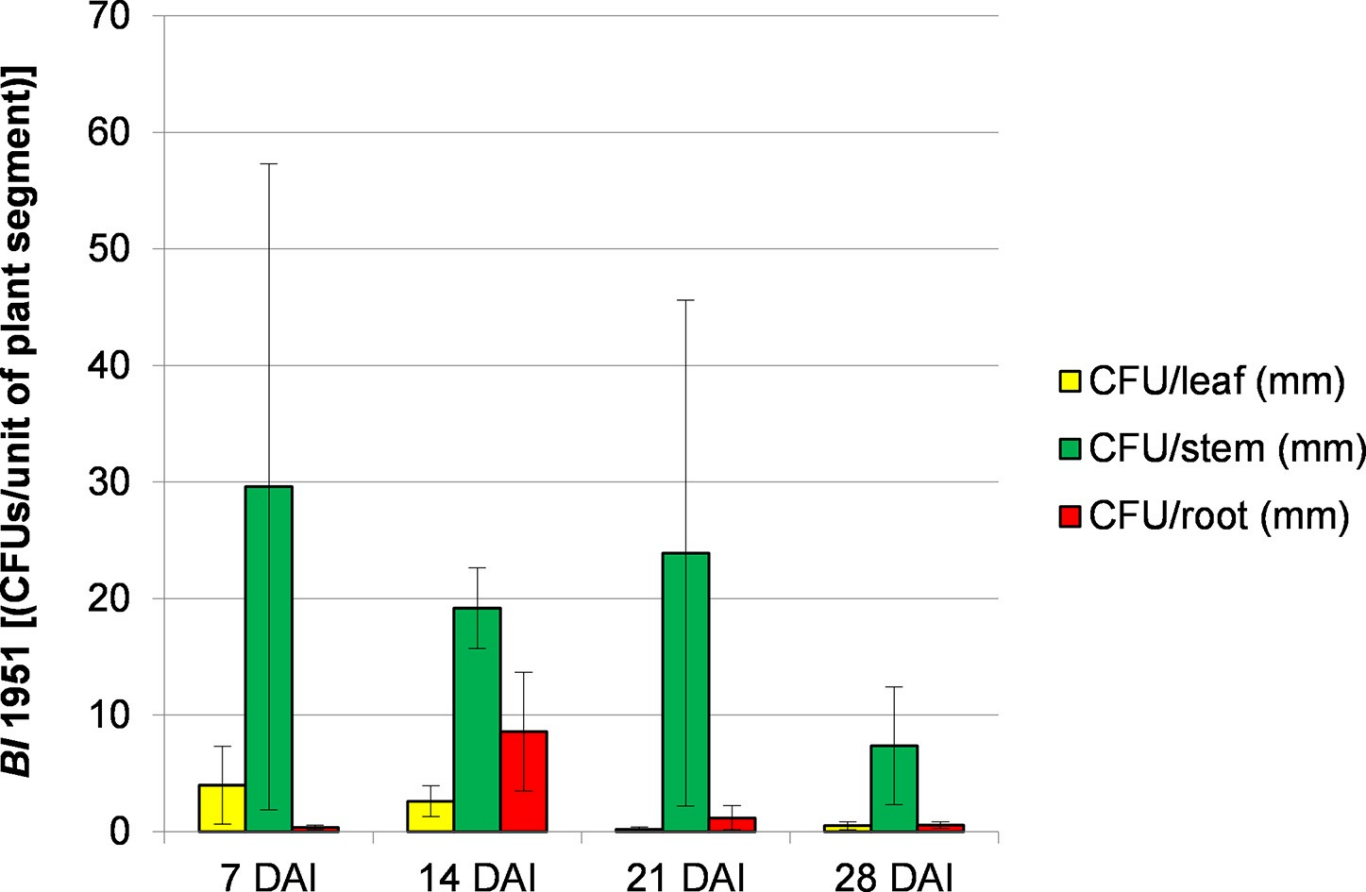
Average colony forming units of *Brevibacillus laterosporus* 1951 per unit of cabbage plant segments. The vertical bars represent the standard errors.

The colony forming units (CFUs) of *Bl* 1951 in different parts of the cabbage plant were determined to quantify the putative endophytic growth within plants. *Bl* 1951 was mostly present in low numbers in cabbage up to 28 DAI, with generally a low CFU/plant segment (Fig 2). The highest quantity of the bacterium was consistently observed within the cabbage stems throughout the sampling dates. Results of this experiment suggest that the bacterium has spread from the roots to stems and leaves. However, the proliferation of the bacterium in each plant part was very low as indicated in the CFU/plant segment.

### 3.3 Recovery of *Brevibacillus laterosporus* 1951 from other brassica species

*Bl* 1951 was recovered from Chinese kale, oilseed rape and radish. *Bl*-like colonies, characterised by serrated edges and creamy in color, were recovered from surface-sterilised leaves, stems and roots samples inoculated with vegetative cells of 1951 from seven DAI until 27 DAI (S7 Fig). The identity of the recovered colonies was confirmed to be that of *Bl* 1951 by sequence analyses through amplification of 16S rDNA and *cry*35-like gene regions.

**Fig 3:**
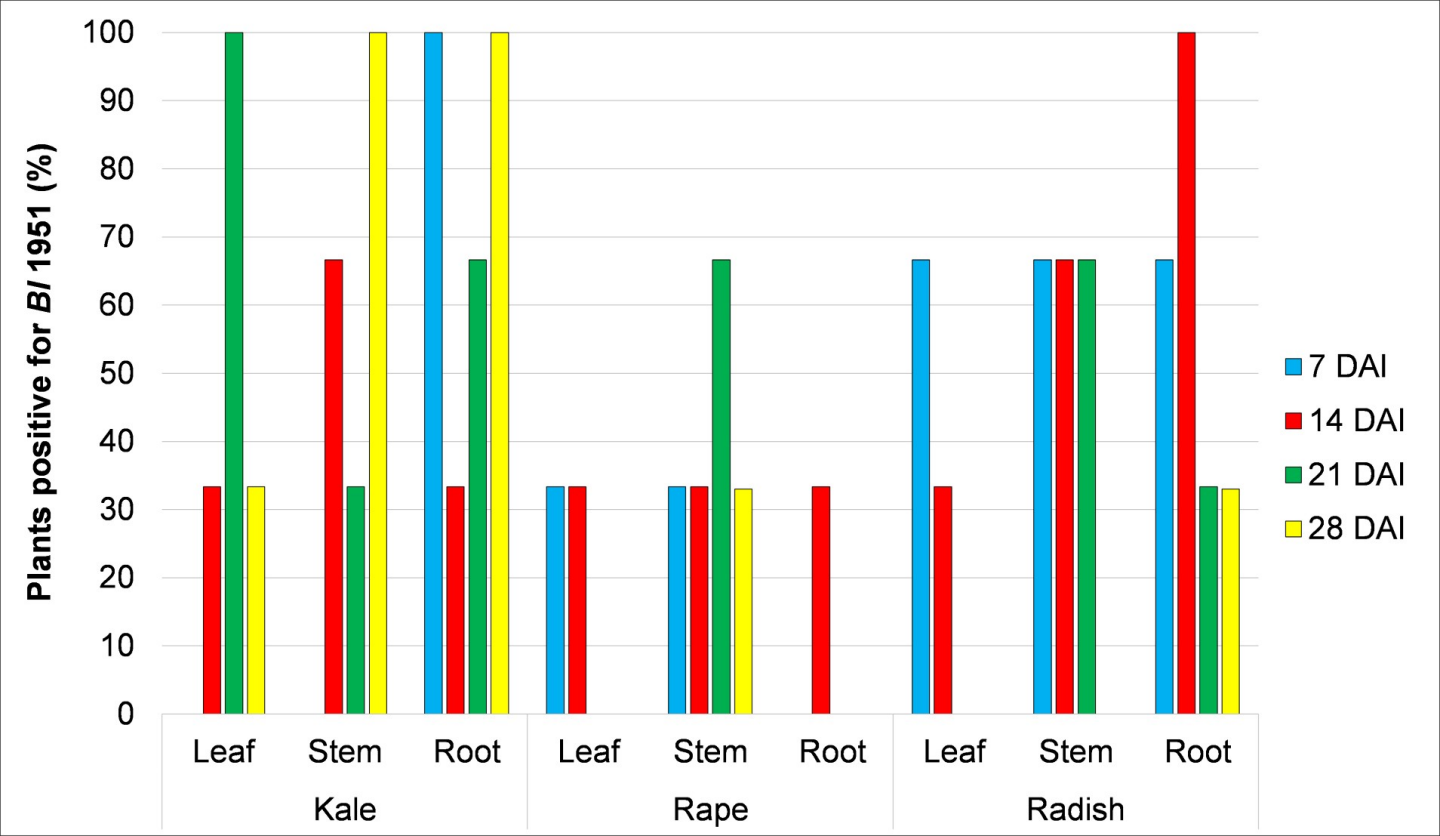
Percentage of brassica plants positive for *Brevibacillus laterosporus* 1951 colonies. Colonies were recovered from different plant parts of Chinese kale, oilseed rape and radish at 7, 14, 21 and 28 days after incubation (n = 3).

The percentage of plants in which the *Bl* 1951 colonies were recovered from different plant parts and sampling days varied between the three *Brassica* species (Fig 3). In general, more *Bl* 1951 was recovered from Chinese kale than oilseed rape and radish, although these were not tested in a single randomised experiment. There was no general trend regarding the persistence of colonisation over time in each plant part among the three species. However, *Bl* 1951 was recovered at each sampling time from the roots of Chinese kale and radish and from the stems of oilseed rape. Recovery of the bacterium from the leaves was inconsistent in all brassica species. In general, control plants were larger compared to the plants inoculated with *Bl* 1951, which was similar to the observations noted in the earlier cabbage pot trials. The plant sizes were not quantified, however.

### 3.4 Recovery of *Brevibacillus laterosporus* 1951 from field-grown cabbage (*Brassica oleracea* var. *capitata*) plants

Ten-week-old cabbage plants from a field sprayed three times with *Bl* 1951 spores over six weeks were tested for endophytic presence of the bacterium. Fifty percent of the sampled plants treated with *Bl* 1951, representing eight out of sixteen plants, were unambiguously positive for *Bl* 1951. One hundred and thirty-two potential endophytic DNA samples, derived from the negative control and *Bl* 1951 treated plants, were screened by PCR. Of these, 13.6%, representing eight plants, were positive for both *cry*27-like and *cry*35-like genes, indicating that they were *Bl* 1951 (Table 1). No *cry*-like genes were detected in the DNA-samples derived from the negative control. The *cry*-like genes that were identified were all derived from potential endophytic bacterial colonies recovered from *Bl* 1951 treated cabbage plants.

**Table 1:** The detection of *cry*27-like and *cry*35-like genes by PCR in the total number of potential endophyte DNA samples, derived from *Brevibacillus laterosporus* 1951 and untreated control treated plants.

Gene present in sample	Percentage detected	Percentage of *Bl* 1951 detected per plant part		
		Roots	Heart leaves	Outer leaves
*cry*27-like & *cry*35-like genes	12.9	5.9	17.6	76.5
*cry*27-like gene	2.3	-	-	-
*cry*35-like gene	3.0	-	-	-
Negative	81.8	-	-	-

The majority of *Bl* 1951 was recovered from the outer leaves, and the least was recovered from the root samples (Table 1). The results suggest that *Bl* 1951 spores may be capable of germinating and colonizing the cabbage plant tissue and potentially live endophytically within the plant after being applied to the plant surface by foliar spray application.

### 3.5 Bioassays with cabbage plants inoculated with *Brevibacillus laterosporus* 1951 against *Plutella xylostella* larvae

The effect of *Bl* 1951 as an endophyte of cabbage plants on DBM larval mortality and pupation was assessed in three separate bioassays with cabbage seedlings of different ages.

**Fig 4:**
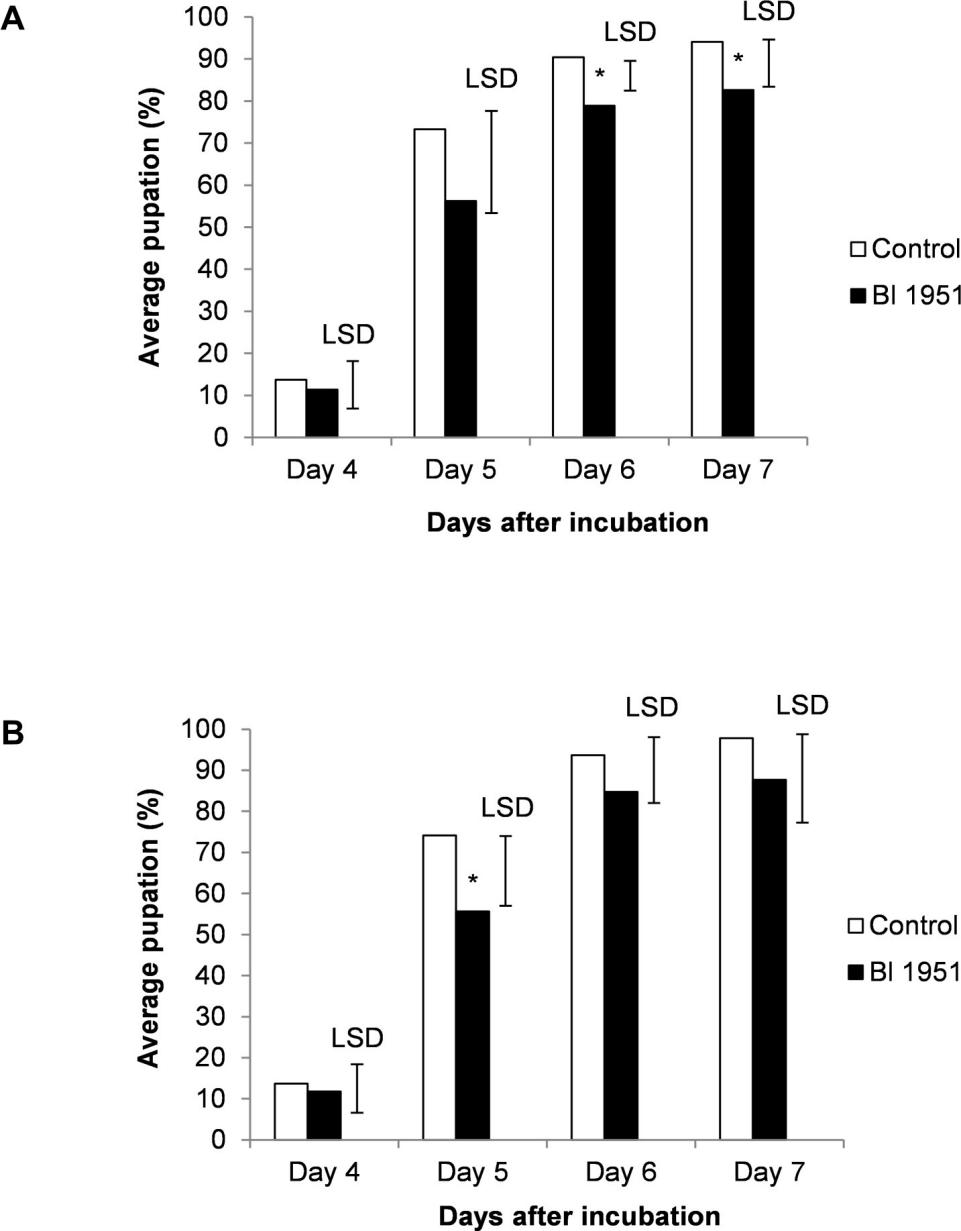
Average pupated diamondback moth larvae of combined bioasssays 1 to 3. Vertical bars are one-sided 5% LSD values (see Methods). (A) Percentage of pupae based on the total number of insects. The average pupation was significantly lower for *Bl* 1951 treated plants on days 6 and 7, with one-sided P-values of 0.021 and 0.049, respectively. (B) Percentage of pupae based on the number of live insects. Plants treated with *Bl* 1951 had a significantly lower average pupation on day 5, with an one-sided P-value of 0.043.

A low percentage cumulative larval mortality was observed in larvae fed leaves from the negative control and *Bl* 1951 inoculated plants in all bioassays (S8 Fig), indicating the absence of a direct effect on DBM larval mortality. No significant differences were detected in the average pupation based on the total number of insects in all three bioassays (S9A, S9C and S9F Fig). However, in the combined bioassays data for *Bl* 1951 treated plants had a significantly lower level of pupation than the negative control on days 6 and 7 after incubation (Fig 4A). A significant difference in the average pupation based on the number of live insects was found in bioassays 1 and 3 (S9B and S9F Fig, respectively), where feeding on *Bl* 1951 treated plants had a significantly lower pupation rate compared to the negative control. Additionally, the combined bioassay data also showed a significantly lower pupation on *Bl* 1951 treated plants on day 5 after incubation (Fig 4B). These results suggest a potential effect of the bacterium presence that delayed the pupation of the DBM larvae.

### 3.6 Confocal microscopy observations of *Brevibacillus laterosporus* 1951 colonisation within cabbage plants

**Fig 5:**
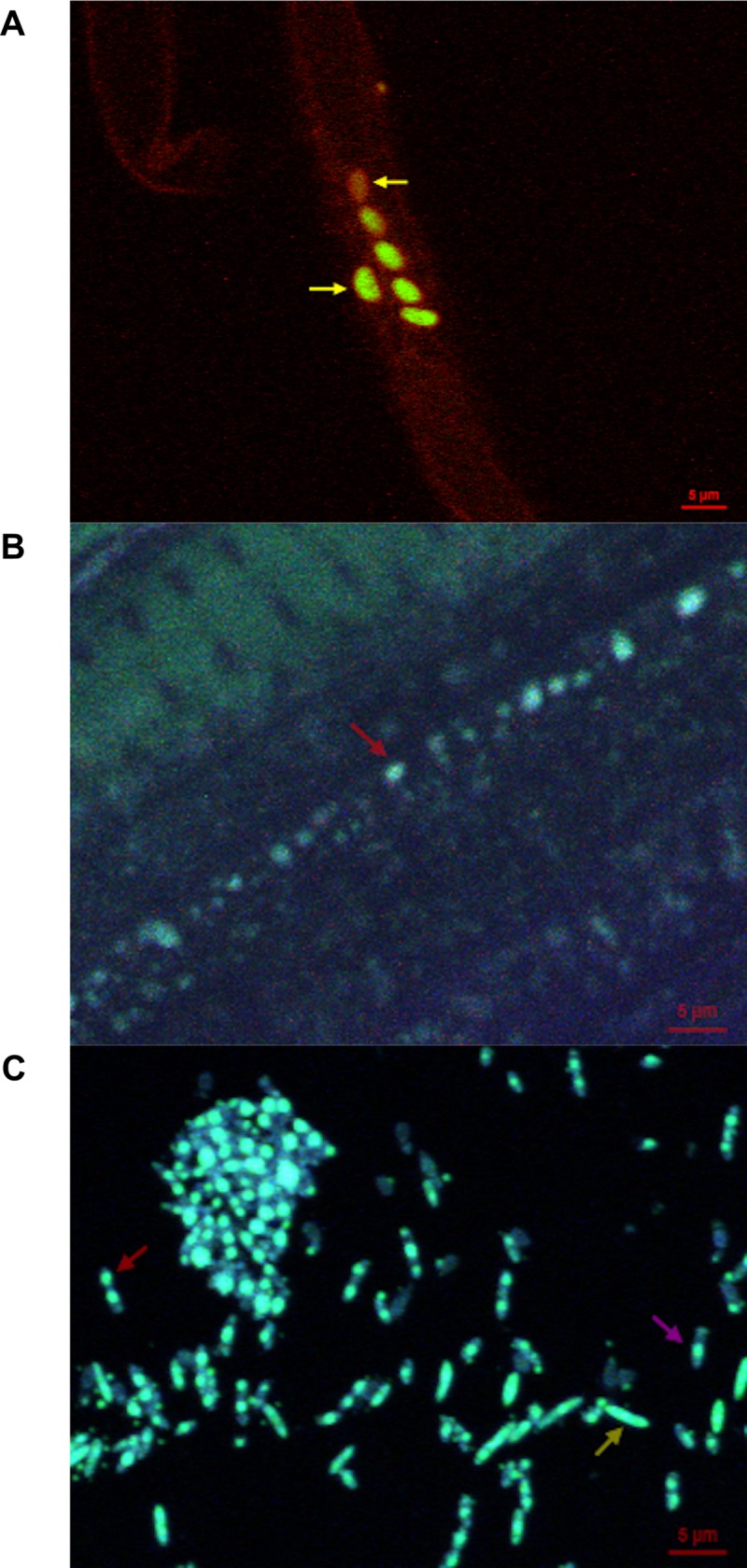
Confocal microscopic images of *Brevibacillus laterosporus* 1951 spores inside the cabbage root tissues. Magnification is 63,000 X and the scale bar size is 5 μm. Red arrows indicate *Bl* 1951 spores. Yellow arrows indicate *Bl* 1951 vegetative cells, and the violet arrow indicates a *Bl* 1951 sporangium. (A) Seven days after inoculation. The top yellow arrow shows a *Bl* cell inside the root tissue. (B) Twenty-one days after inoculation. (C) *Bl* 1951 sporulated culture as a positive control.

Brightly fluorescing *Bl* 1951 cells were observed in the root tissues at 7, 14, and 21 DAI. The fluorescing cells resembled vegetative *Bl* cells. Some of the cells appeared to be embedded inside the tissues (Fig 5A), while some were found along the periphery of the cell wall (Fig 5B). The size of the fluorescing cells approximated the size of vegetative cells and spores in the stained, sporulating culture (Fig 5C). The fluorescing cells were few in number, indicating low colonisation, which is consistent with the low CFU of *Bl* recovered from the different cabbage plant parts. No *Bl* cells or spores were observed in the negative control.

## 4. Discussion

Endophytes are defined as “microbes which occur within plant tissue for at least a part of their life cycle without causing disease under any known circumstances” [22]. Plants experience abiotic and biotic stresses during their lifetime [23]. Some fungal and bacterial endophytes have been shown to reduce the impact of such stresses in laboratory conditions [22, 24–27].

*Bl* isolates 1821 and 1951 were recovered as potential endophytes from cabbage seedlings that had the roots treated with the vegetative cells of both *Bl* strains. The spread of *Bl* 1951 from the roots through stems to leaves was confirmed by the evaluation of colony forming units. *Bl* 1951 was also recovered from other brassica species tested (Chinese kale, oilseed rape and radish). Additionally, *Bl* 1951 was recovered as a putative endophyte from mature cabbage plants in the field, which had been sprayed with *Bl* 1951 spores. Putative *Bl* 1951 vegetative cells and spores were observed in and at the periphery of the cabbage root tissue by confocal microscopy, confirming the endophytic potential of this strain. The results suggest that both strains may be capable of living as endophytes in cabbage and other brassicas. This is the first report of *Bl* species as potential endophytes.

The possible endophytic ability of *Bl* 1821 and 1951 suggests that the strains may be ingested during human consumption of cabbages treated with these strains. Therefore, it is essential to ascertain that both strains are safe for human use before development as an invertebrate control agent. Organisation for Economic Co-operation & Development (OECD) standard toxicity tests were performed on rats with *Bl* 1821 and 1951 without causing any adverse effects on these small mammals [28]. Additionally, a strain of *Bl* has been registered as a commercial probiotic product for human consumption [29] and another *Bl* strain has been patented for use as a probiotic for poultry [30].

Plant growth can be inhibited or reduced by various abiotic and biotic stresses such as extreme temperatures, soil pH, flooding, drought, insect herbivory and pathogens [23]. *Bl* 1821 and 1951 have demonstrated significant protection against DBM larval herbivory of cabbage plants under laboratory and field conditions when topically applied as cells or spores. As a potential endophyte, however, *Bl* 1951 showed no limiting effect on DBM larval herbivory. This may be linked to the low *Bl* 1951 CFUs that were recovered from the roots, stems and leaves of cabbage seedlings during this study. In other studies, high doses of spores and cells were used in the laboratory trials where significant DBM larval mortality, up to 100%, has been observed with *Bl* 1821 and 1951 topically treated plants [3] (S10 Fig). When diluted doses of sporulated cultures were used, little effect on larval mortality was observed (S10 Fig). Although *Bl* 1951 as a potential endophyte provided no protection against DBM larval herbivory, it did slightly reduce DBM pupation and may provide protection against other biotic plant stresses, e.g. plant pathogens. The bacterium may have an effect on abiotic plant stresses as well, as this has been reported with other endophytes [24, 25, 27].

The cabbage seedlings treated with *Bl* appeared to grow slower than the seedlings of the negative control in the endophyte pot trials. These results suggest that *Bl* 1821 and 1951 may have a slightly inhibitory effect on the growth of cabbage plants, which may be a consequence of the putative endophytic colonisation of the strains.

Indirect plant growth promotion by endophytes can be achieved by the inhibition of antagonistic substances normally produced by the host plant against bacterial or fungal pathogens, in other words by deactivating the plant’s systemic immune response [23, 31, 32]. It could, therefore, be argued that promoting the plant’s systemic immune response may have an inhibitory effect on plant growth. The protein pEBL1, derived from *Bl* A60, was found to trigger a hypersensitivity response (HR) and systemic resistance in the tobacco plant (*Nicotiana benthamiana*) when heterologously expressed [33]. *Bl* 1821 and 1951 may trigger plant defense systems in brassicas as well, which may offer protection, but may have a slowing effect on plant growth consequently. Alternatively, *Bl* 1821 and 1951 may affect plant growth in another way.

Weise et al. [34] demonstrated that high levels of bacterial ammonia volatiles from *Serratia odorifera* 4Rx13 and six other rhizobacteria had a significant inhibitory effect on the growth of *Arabidopsis thaliana*. Over 55 putative ammonia-producing enzyme encoding genes were found in the *S*. *odorifera* genome [34]. *Bl* 1821 and 1951 may produce ammonia volatiles as well, which may have an inhibitory effect on plant growth.

The majority of endophytes originate from the rhizosphere [23], a narrow zone of soil that is directly influenced by root exudates and by the microbes that inhabit there [35–40]. Given that the roots of the cabbage seedlings were dipped in the vegetative cells of *Bl* 1821 and 1951 during the endophyte pot trial, it is likely that the strains entered the internal plant tissue via the roots. Other points of entry for endophytic colonisation include the stomata, especially on leaves and young stems, germinating radicles [23, 41], and lenticels, which are generally present in the periderm of stems and roots [23, 42].

During the field trial, spores of *Bl* 1951 were sprayed onto the surface of cabbage plants. Some of these spores may have entered the internal plant tissue through the stomata present on the leaves and stems, subsequently germinated and migrated to other parts of the plant tissue. Additionally, it is possible that a fraction of the sprayed spores ended up in the soil and were further absorbed into the soil due to irrigation and rain showers. Some of these spores may have reached the plant’s rhizosphere and colonised the internal plant tissue via the roots. The root exudates trigger chemotactic movement of bacteria towards them [23, 43]. Consequently, the root exudates of cabbage plants used in the field trial may have contained compounds that triggered the germination and chemotactic movement of any *Bl* 1951 spores that may have reached the plant’s rhizosphere after spray application.

Colonisation of the endophyte inside the plant tissues can be confirmed through microscopic observation of micro sections or indirectly by measurement of the colony forming units per plant part. Results of this experiment suggest that the bacterium has spread from the roots to stems and leaves. However, the proliferation of the bacterium in each plant part was low as indicated in the CFU/plant segment.

The surface-sterilised untreated control plants of the first cabbage endophyte pot trial contained colonies after being incubated for seven days on selective agar. As mentioned previously, the scalpel that was used to cut the plant parts after surface sterilisation and before incubation on selective agar may have been contaminated. Extra care was taken in the following three weeks to decontaminate the scalpels thoroughly in between samples.

The confocal images of *Bl* suggest that the cells had colonised cabbage inner root tissue by 7 DAI, as one cell was clearly visible inside the tissue. At 21 DAI, spores were observed at the periphery of the roots, suggesting an epiphytic nature of the cells as well. However, the identity of the cells could not be definitely confirmed. To unequivocally confirm *Bl*’s presence inside plant tissue by confocal microscopy, the cells need to be transformed to express green fluorescent protein (GFP) or another fluorescent protein.

With the results of the laboratory and field experiments demonstrating *Bl* isolates 1821 and 1951 can become endophytic, the next steps to pursue are to evaluate *Bl*- inoculated cabbage seedlings against other biotic and abiotic stresses such as pathogens, drought and resistance to extreme temperatures. *Bl*, as a potential endophyte, could be a valuable strategy in improving cabbage and other brassica production by enhancing the plant’s defense mechanism against biotic and abiotic stresses.

## Supporting information

S1 Fig Schematic overview of the endophyte pot trials design and the field trial design. (A) Experimental design of the endophyte pot trial with cabbage seedlings and *Brevibacillus laterosporus* 1821 and 1951. Numbers: 1, 2, 3 and 4 represent the weeks when the respective plants were harvested for surface sterilisation. For example: 1 = week one. 2 = week two, etc. Each replicate was located on a separate shelf within the incubator. Treatments were not randomised within replicates, but sampling weeks were randomised within each sterile container. Abbreviation: Rep = replicate.
(B) Overview of the field trial design from which the cabbages of the negative control and treated with *Brevibacillus laterosporus* 1951 were recovered for the detection of endophytes. The red circles indicate the plants that were harvested and sampled for the endophyte detection of *Bl* 1951. Abbreviations: Brevi = *Brevibacillus laterosporus*;—Control = Negative control; Rep = replicate.
(TIFF)

S2 Fig ***Brevibacillus laterosporus* 1821 (B, E, H) and 1951 (C, F, I) growing on semi-selective agar, from surface sterilised cabbage seedling tissues 7 days after inoculation.** Control seedlings (A, D, G) were free of *Bl*. The red arrows indicate bacterial colonies.
(TIFF)

S3 Fig Phylogenetic tree of 16S rDNA sequences of the negative control samples recovered from cabbage seedlings 7 days after incubation. Formatted in Geneious [21]. The scale bar represents the nucleotide substitutions per site (the number of changes per 100 nucleotide sites). The red bracket indicates the position of the negative control potential endophyte samples and the *Bl* 1821 and 1951 positive controls (+). Abbreviations: B. = *Bacillus*; Br. = *Brevibacillus*.
(TIFF)

S4 Fig ***Brevibacillus laterosporus* 1821 (B, E, H) and 1951 (C, F, I) growing on semi-selective agar, from surface sterilised cabbage seedling tissues 14 days after inoculation.** Control seedlings (A, D, G) were free of *Bl*. The red arrows indicate bacterial colonies.
(TIFF)

S5 Fig ***Brevibacillus laterosporus* 1821 (B, E, H) and 1951 (C, F, I) growing on semi-selective agar, from surface sterilised cabbage seedling tissues 23 days after inoculation.** Control seedlings (A, D, G) were free of *Bl*. The red arrows indicate bacterial colonies.
(TIFF)

S6 Fig ***Brevibacillus laterosporus* 1821 (B, E, H) and 1951 (C, F, I) growing on semi-selective agar, from surface-sterilised cabbage seedling tissues 28 days after inoculation.** Control seedlings (A, D, G) were free of *Bl*. The red arrows indicate bacterial colonies growing from the surface-sterilised plant tissue.
(TIFF)

S7 Fig Surface sterilised leaf, stem and root samples of Chinese kale on semi-selective medium showing *Brevibacillus laterosporus*-like colonies. Abbreviation: DAI = days after inoculation.
(TIFF)

S8 Fig Cumulative mortality of diamondback moth larvae of combined bioasssays 1 to 3. Vertical bars are one-sided 5% LSD values (see Methods). There were no signficant differences between the treatments from day 1 to day 7.
(TIF)

S9 Fig Average percentage pupated diamondback moth larvae of bioassay repeats 1, 2 and 3. Plant ages were 18, 27 and 23 days for bioassay repeat 1, 2 and 3, respectively. Vertical bars are one-sided 5% LSD values (see Methods).
(A) Percentage of pupae based on the total number of insects, bioasay repeat 1.
(B) Percentage of pupae based on the number of live insects, bioassay repeat 1. The average pupae number was significantly lower for *Bl* 1951 treated plants on days 6 and 7, with one-sided P-values of 0.048 and 0.040, respectively.
(C) Percentage of pupae based on the total number of insects, bioassay repeat 2.
(D) Percentage of pupae based on the number of live insects, bioassay repeat 2.
(E) Percentage of pupae based on the total number of insects, bioassay repeat 3.
(F) Percentage of pupae based on the number of live insects, bioassay repeat 3. The average pupae number was significantly lower for *Bl* 1951 treated plants on day 7, with an one-sided P-value of 0.019.
(TIFF)

S10 Fig Bioassay against diamondback moth larvae with *Brevibacillus laterosporus* 1951 sporulated cultures, cultured in different conditions. General ANOVA, days 2 and 3, LSD = 5%. All full strength cultures had a significantly higher cumulative mortality compared to the negative control on day 2 (P = 0.004). Only full strength Culture 1 had a significantly higher mortality compared to the negative control on day 3 (P = 0.007).
(TIF)

S1 Data Fasta file containing the 16S rDNA sequence alignment for the Fig 1 phylogenetic tree. (FASTA)

S2 Data Excel file containing the underlying data for Fig 2. (XLSX)

S3 Data Excel file containing the underlying data for Fig 3. (XLSX)

S4 Data Excel file containing the underlying data for Fig 4, S8 and S9 Figs. (XLSX)

S5 Data Excel file containing the underlying data for Table 1. (ZIP)

S6 Data Fasta file containing the 16S rDNA sequence alignment for the S3 Fig phylogenetic tree. (FASTA)

S7 Data Excel file containing the underlying data for S10 Fig. (XLSX)
