# Supplementary figures and images for: Endophytic ability of the insecticidal bacterium Brevibacillus laterosporus in Brassica
Source: PLoS One. 2019 May 22;14(5):e0216341. doi: 10.1371/journal.pone.0216341 (PMC6530831; doi:10.1371/journal.pone.0216341)

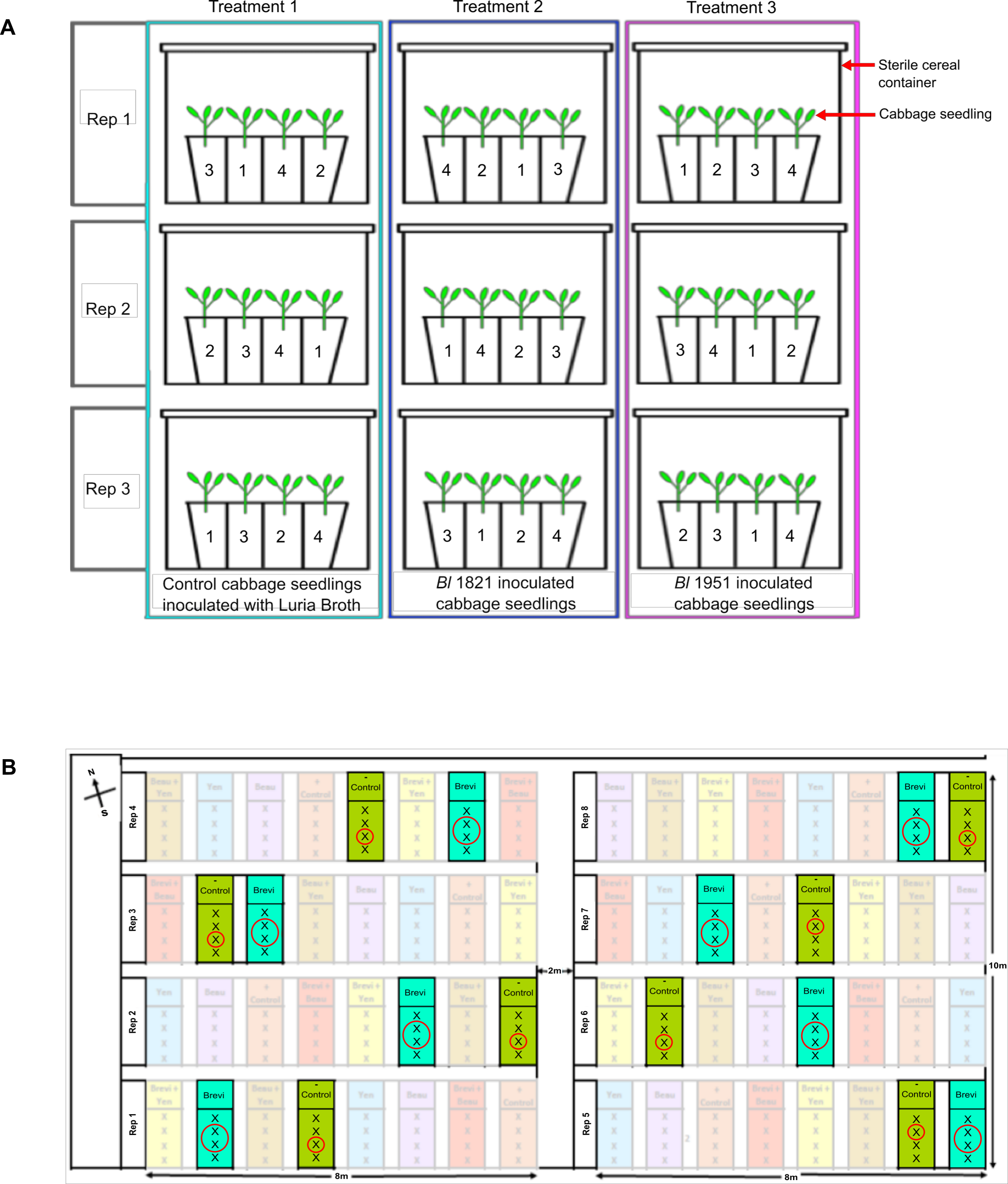

Supplement: S1 Fig — (A) Experimental design of the endophyte pot trial with cabbage seedlings and Brevibacillus laterosporus 1821 and 1951. Numbers: 1, 2, 3 and 4 represent the weeks when the respective plants were harvested for surface sterilisation. For example: 1 = week one. 2 = week two, etc. Each replicate was located on a separate shelf within the incubator. Treatments were not randomised within replicates, but sampling weeks were randomised within each sterile container. Abbreviation: Rep = replicate. (B) Overview of the field trial design from which the cabbages of the negative control and treated with Brevibacillus laterosporus 1951 were recovered for the detection of endophytes. The red circles indicate the plants that were harvested and sampled for the endophyte detection of Bl 1951. Abbreviations: Brevi = Brevibacillus laterosporus;—Control = Negative control; Rep = replicate. (TIFF) [file pone.0216341.s001.tiff]

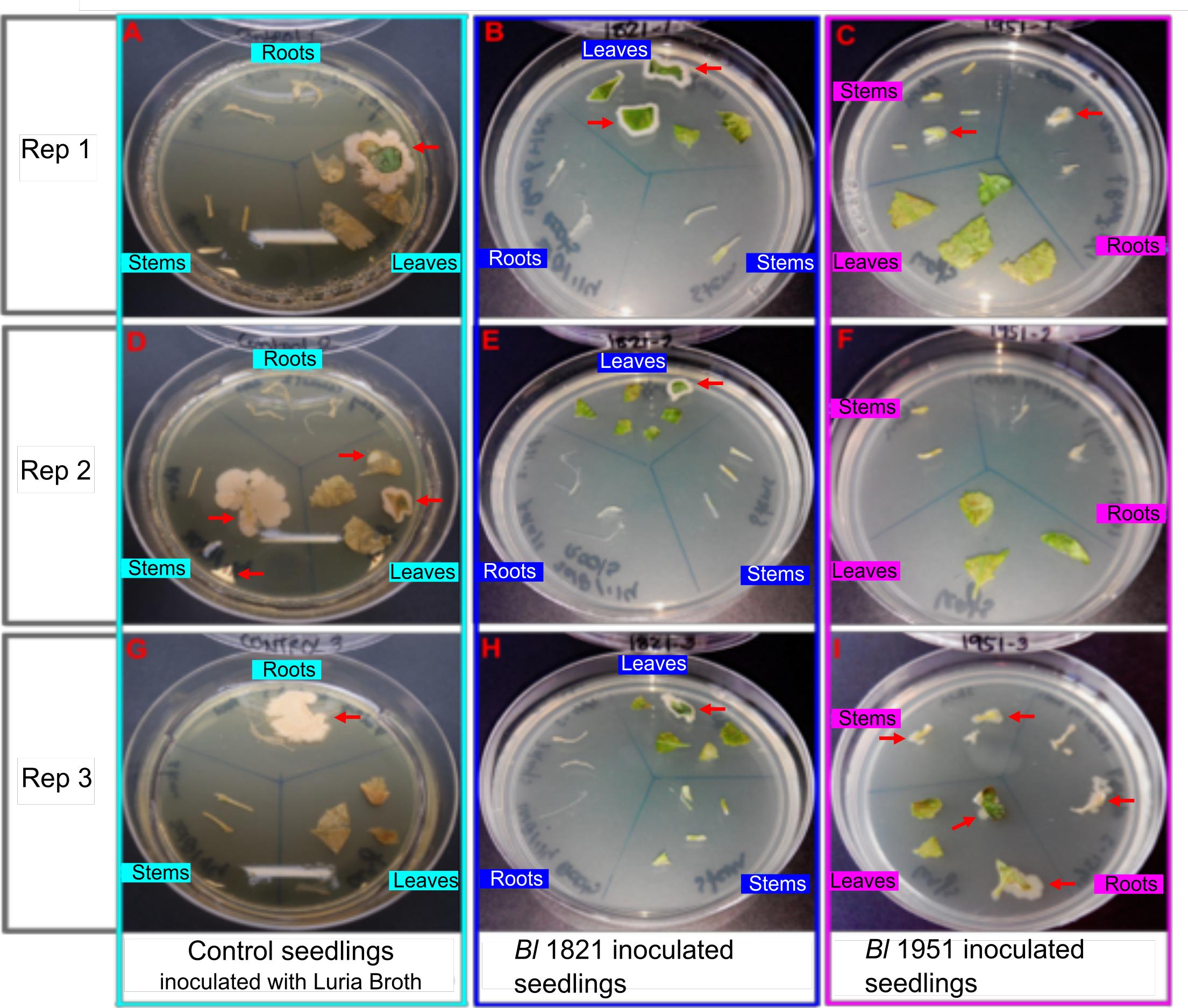

Supplement: S2 Fig — Brevibacillus laterosporus 1821 (B, E, H) and 1951 (C, F, I) growing on semi-selective agar, from surface sterilised cabbage seedling tissues 7 days after inoculation. Control seedlings (A, D, G) were free of Bl. The red arrows indicate bacterial colonies. (TIFF) [file pone.0216341.s002.tiff]

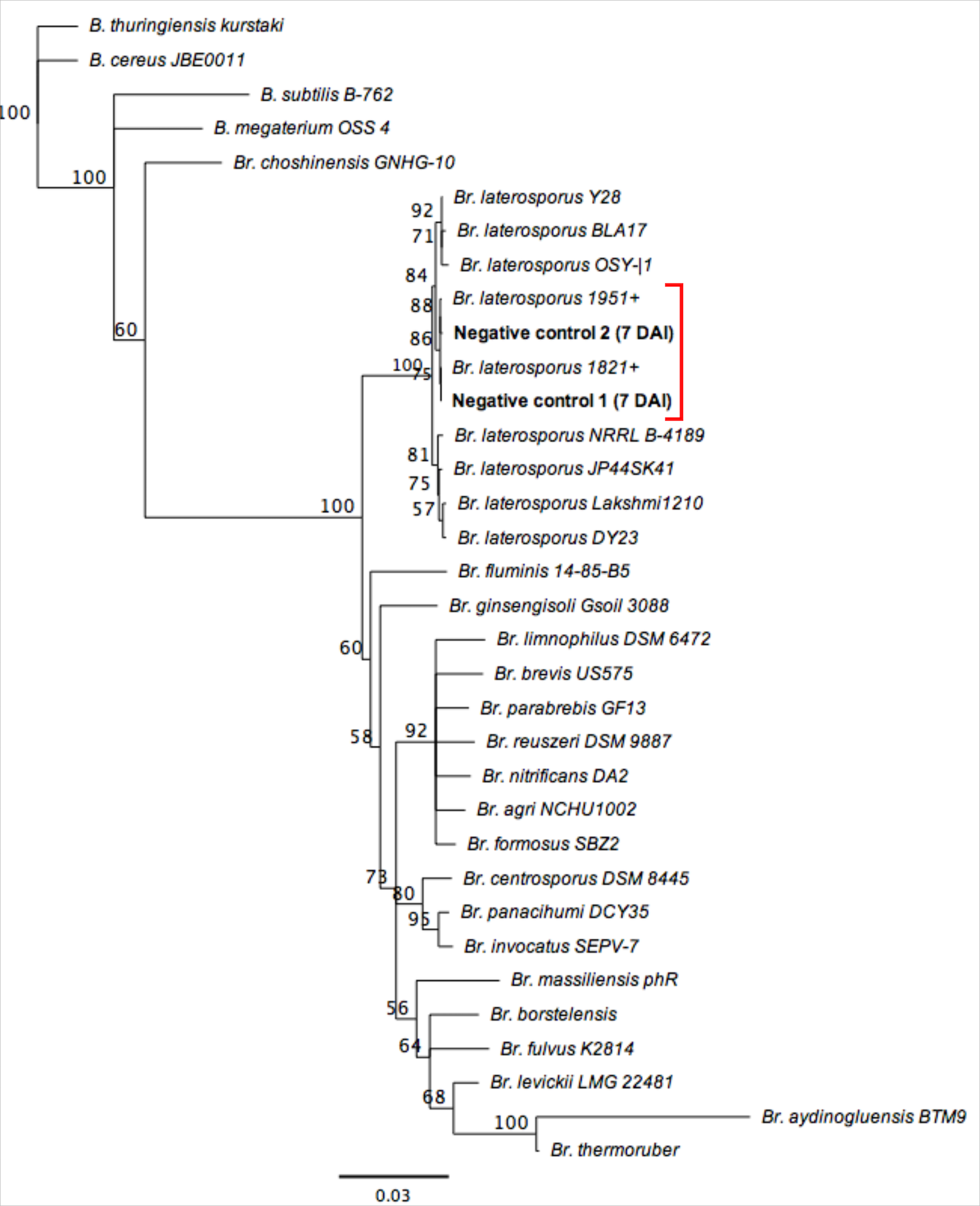

Supplement: S3 Fig — Formatted in Geneious [21]. The scale bar represents the nucleotide substitutions per site (the number of changes per 100 nucleotide sites). The red bracket indicates the position of the negative control potential endophyte samples and the Bl 1821 and 1951 positive controls (+). Abbreviations: B. = Bacillus; Br. = Brevibacillus. (TIFF) [file pone.0216341.s003.tiff]

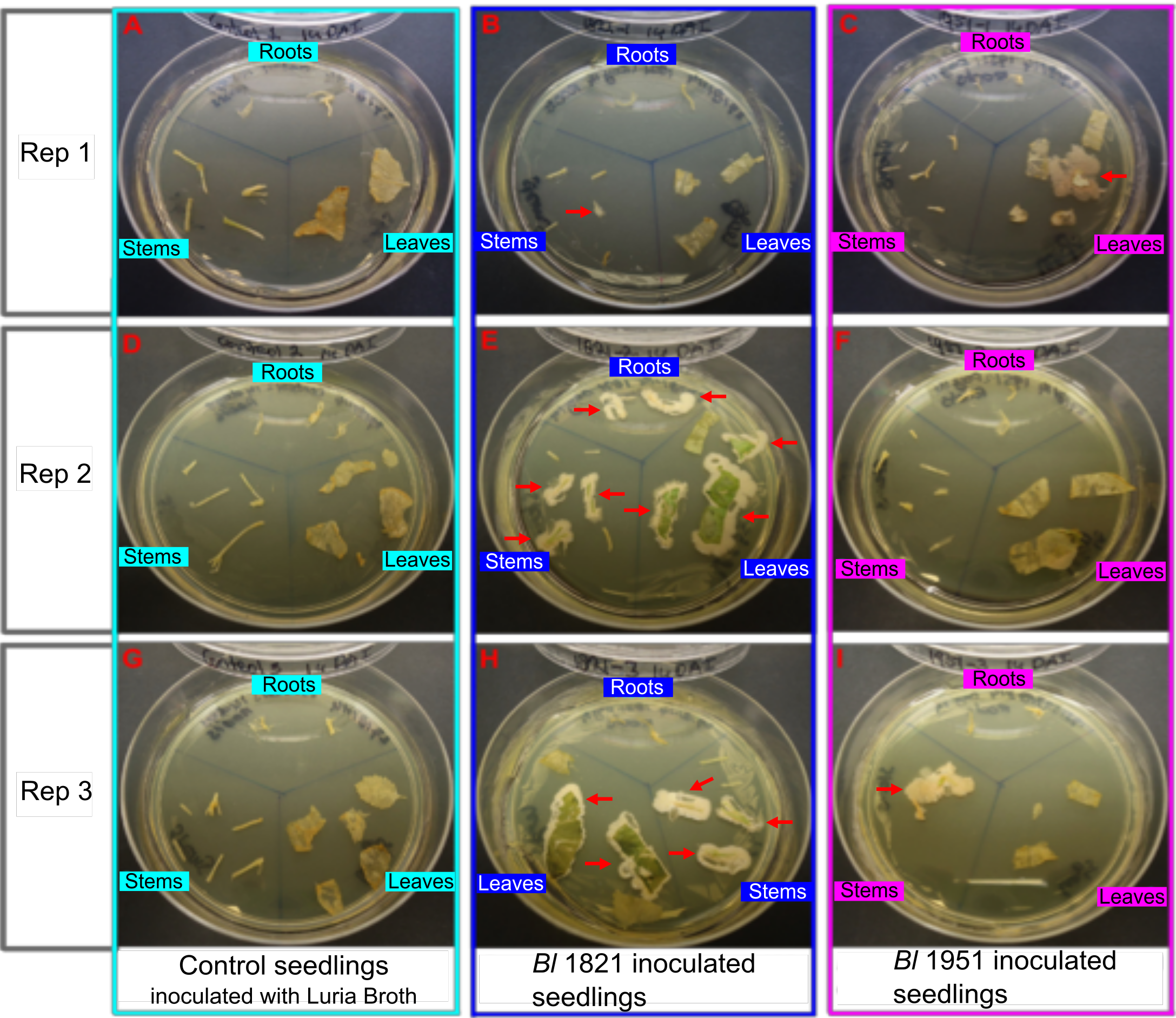

Supplement: S4 Fig — Brevibacillus laterosporus 1821 (B, E, H) and 1951 (C, F, I) growing on semi-selective agar, from surface sterilised cabbage seedling tissues 14 days after inoculation. Control seedlings (A, D, G) were free of Bl. The red arrows indicate bacterial colonies. (TIFF) [file pone.0216341.s004.tiff]

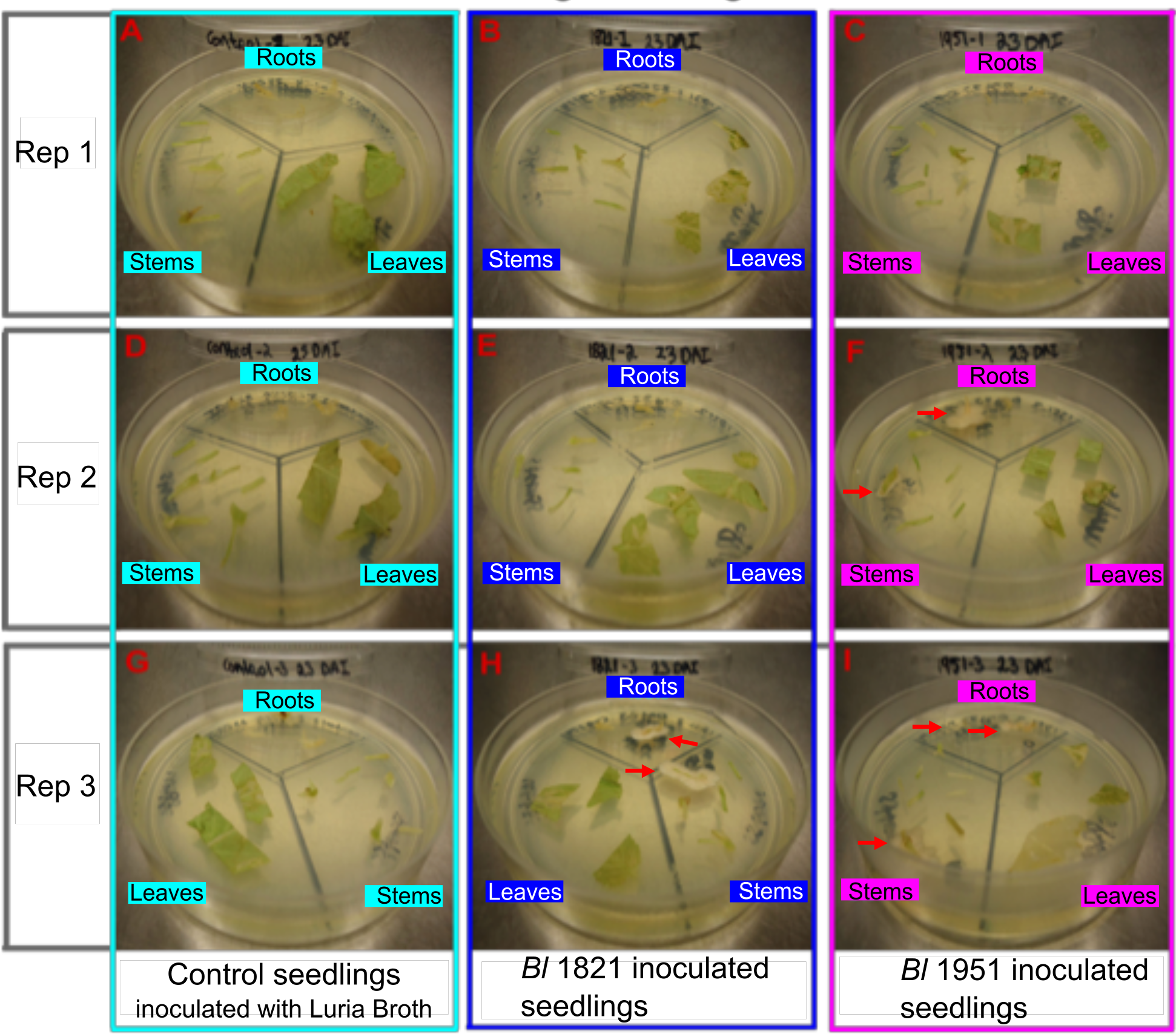

Supplement: S5 Fig — Brevibacillus laterosporus 1821 (B, E, H) and 1951 (C, F, I) growing on semi-selective agar, from surface sterilised cabbage seedling tissues 23 days after inoculation. Control seedlings (A, D, G) were free of Bl. The red arrows indicate bacterial colonies. (TIFF) [file pone.0216341.s005.tiff]

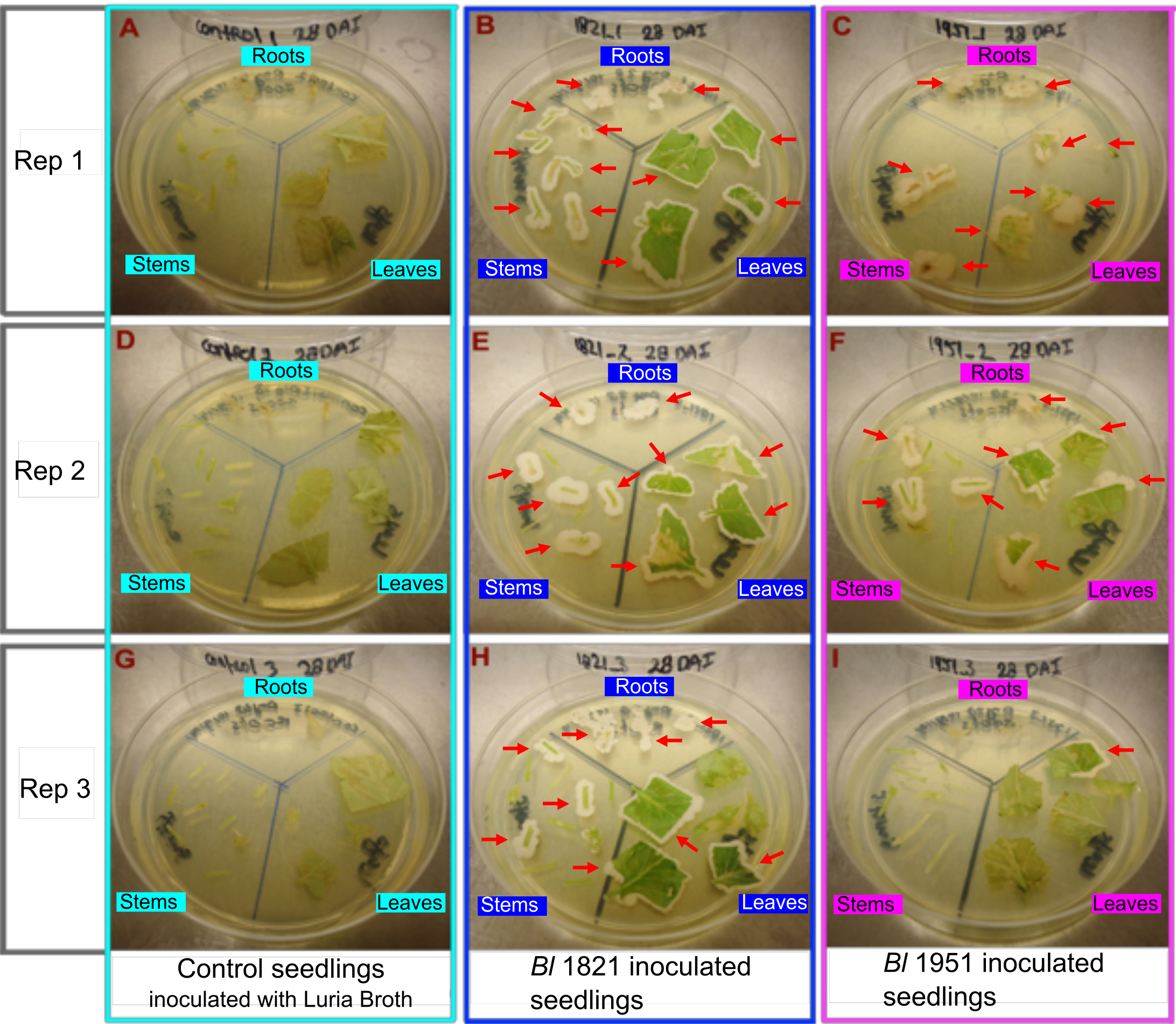

Supplement: S6 Fig — Brevibacillus laterosporus 1821 (B, E, H) and 1951 (C, F, I) growing on semi-selective agar, from surface-sterilised cabbage seedling tissues 28 days after inoculation. Control seedlings (A, D, G) were free of Bl. The red arrows indicate bacterial colonies growing from the surface-sterilised plant tissue. (TIFF) [file pone.0216341.s006.tiff]

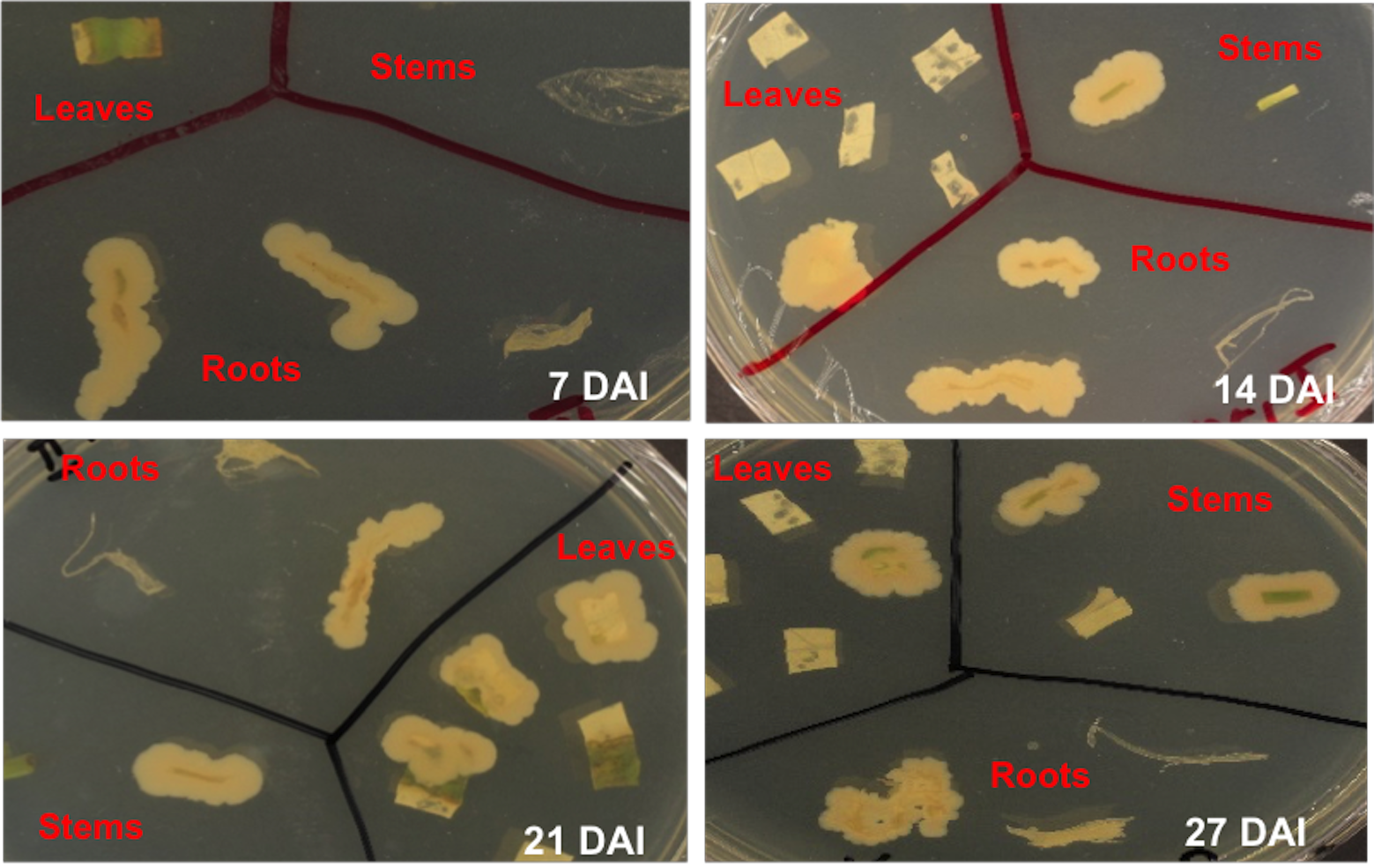

Supplement: S7 Fig — Abbreviation: DAI = days after inoculation. (TIFF) [file pone.0216341.s007.tiff]

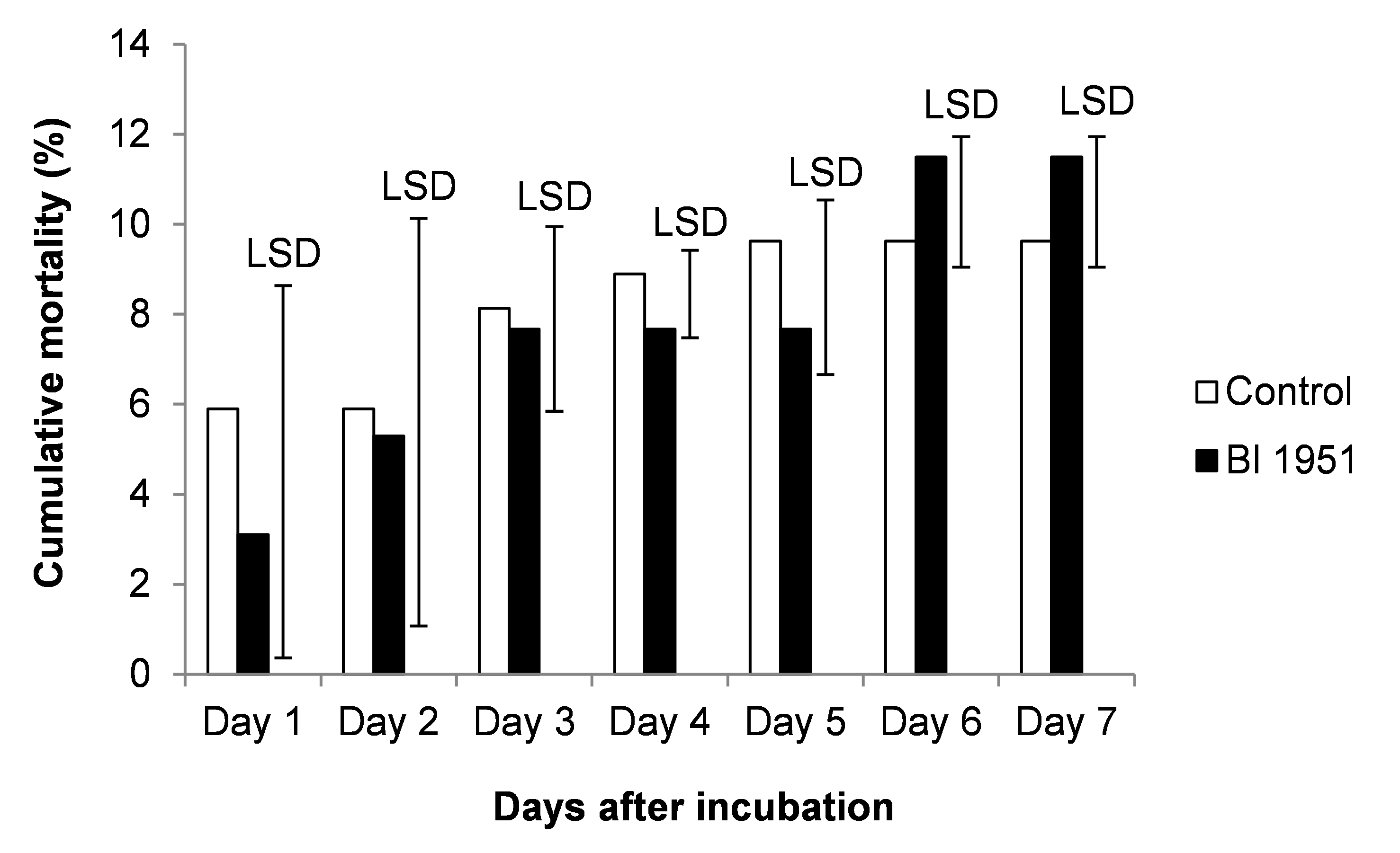

Supplement: S8 Fig — Vertical bars are one-sided 5% LSD values (see Methods). There were no signficant differences between the treatments from day 1 to day 7. (TIF) [file pone.0216341.s008.tif]

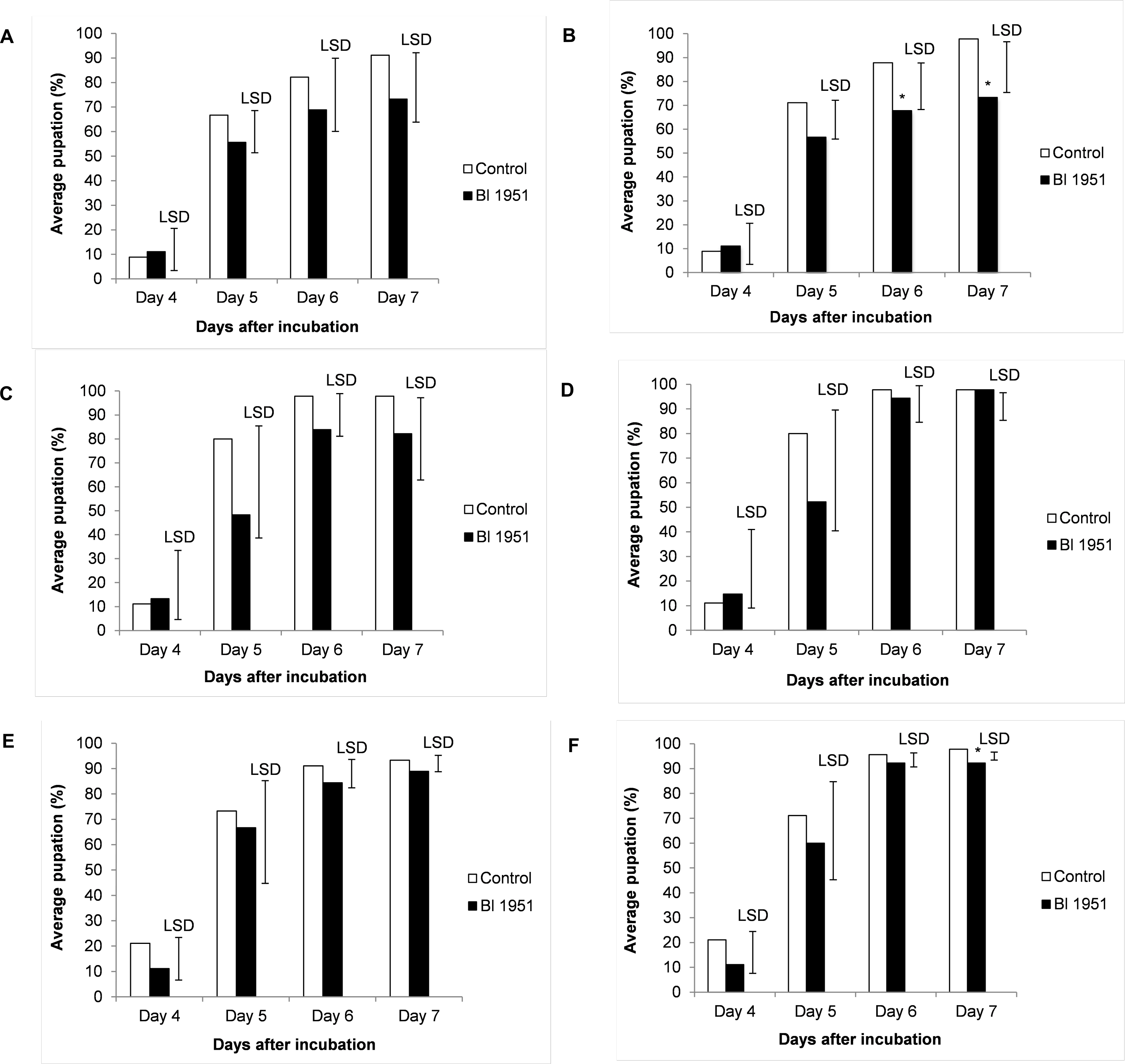

Supplement: S9 Fig — Plant ages were 18, 27 and 23 days for bioassay repeat 1, 2 and 3, respectively. Vertical bars are one-sided 5% LSD values (see Methods). (A) Percentage of pupae based on the total number of insects, bioasay repeat 1. (B) Percentage of pupae based on the number of live insects, bioassay repeat 1. The average pupae number was significantly lower for Bl 1951 treated plants on days 6 and 7, with one-sided P-values of 0.048 and 0.040, respectively. (C) Percentage of pupae based on the total number of insects, bioassay repeat 2. (D) Percentage of pupae based on the number of live insects, bioassay repeat 2. (E) Percentage of pupae based on the total number of insects, bioassay repeat 3. (F) Percentage of pupae based on the number of live insects, bioassay repeat 3. The average pupae number was significantly lower for Bl 1951 treated plants on day 7, with an one-sided P-value of 0.019. (TIFF) [file pone.0216341.s009.tiff]

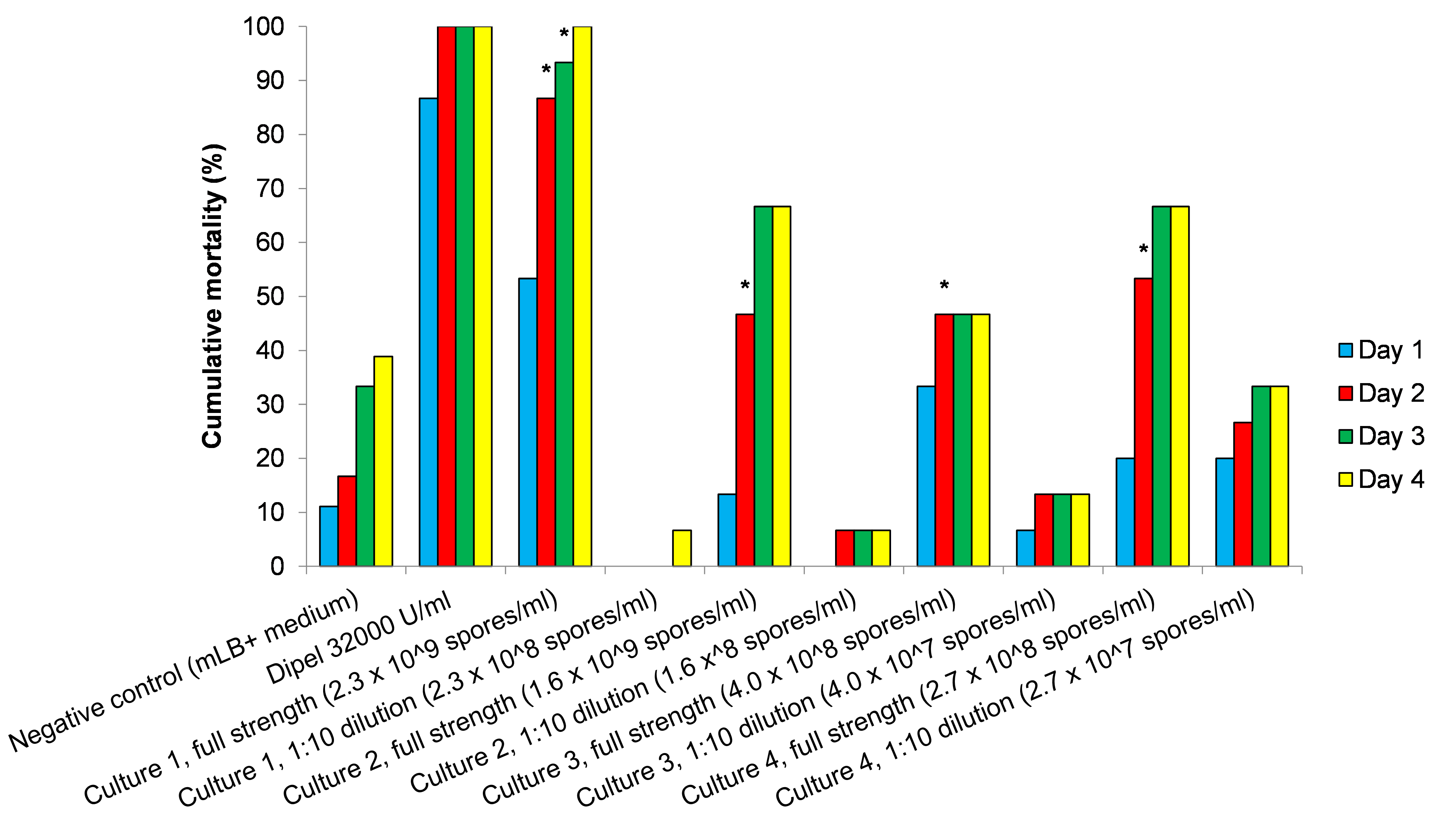

Supplement: S10 Fig — General ANOVA, days 2 and 3, LSD = 5%. All full strength cultures had a significantly higher cumulative mortality compared to the negative control on day 2 (P = 0.004). Only full strength Culture 1 had a significantly higher mortality compared to the negative control on day 3 (P = 0.007). (TIF) [file pone.0216341.s010.tif]
